# Rapid risk assessment to address emerging concerns of HPAI in raw and pasteurized milk

**DOI:** 10.1371/journal.pone.0322948

**Published:** 2025-06-04

**Authors:** Yuhuan Chen, Kara J. Dean, Gavin J. Fenske, Sarah I. Murphy, Alexandra Gavelek, Régis Pouillot, Jane M. Van Doren, Sherri Dennis

**Affiliations:** 1 Human Foods Program, U.S. Food and Drug Administration, College Park, Maryland, United States of America; 2 Goldbelt C6, LLC, Chesapeake, Virginia, United States of America; Universidad Cooperativa de Colombia, COLOMBIA

## Abstract

An outbreak of highly pathogenic avian influenza A, subtype H5N1, first reported in U.S. dairy cattle in March 2024, raised concerns of an emerging food safety threat from the virus in the milk supply. To support potential regulatory responses, we conducted a rapid assessment of the predicted risk to U.S. consumers of cow’s milk with two complementary and parallel approaches: a “bottom-up” quantitative risk assessment model that integrated data on virus levels in milk, milk consumption, and dose response; and a “top-down” epidemiological analysis that linked current novel flu illness detection to the consumption of raw and pasteurized milk. The dynamic use of the approaches accommodated rapidly evolving data in a range of risk scenarios. The risk assessment model identified pasteurization as a critical control for H5N1 in milk and highlighted the need for i) the targeted sampling of bulk tank raw milk in affected states pre-pasteurization, ii) raw milk herd surveillance and sampling, and iii) a better understanding of ingestion as a route of H5N1 infections for humans. This novel approach and the findings from this study promoted informed decision-making in an evolving outbreak investigation. This methodology can be leveraged in the conduct of future risk assessments to address emerging pathogen outbreaks that impact the food supply.

## Introduction

The U.S. Food and Drug Administration (FDA), U.S. Department of Agriculture (USDA), U.S. Centers for Disease Control and Prevention (CDC), and state partners collaborated to investigate and respond to an emerging outbreak of influenza A (H5N1) in U.S. dairy cattle that has infected dairy herds in at least 17 U.S. states (as of February 27, 2025 [1]). H5N1 was detected in U.S. dairy cattle in March 2024, following the examination of animals with an unspecified illness and subsequent testing of milk and other samples [2]. Preliminary phylogenetic analysis places the isolated viruses in a monophyletic clade (common ancestor) with Eurasian subtypes of H5N1 (clade 2.3.4.4b) [2], which has been circulating in the U.S. since at least 2022 [3,4]. While the H5N1 B3.13 strain has infected dairy cattle since March 2024, more recently there were several instances of spillover of the H5N1 D1.1 strain (circulating in wild birds) into the U.S. dairy cattle [5]. H5N1 is classically associated with some avian species, where it is transmitted via direct and indirect contact with respiratory or fecal secretions [6]. While most avian influenza subtypes only generate mild or asymptomatic infections in birds, highly pathogenic avian influenzas contain a unique amino acid motif in the hemagglutinin (protein which binds and facilitates entry into host cells) cleavage site [7,8], which can dramatically increase the severity of disease in some species of birds by allowing for systemic spread of the virus [9].

The detected human infections from the H5N1 subtype of the avian influenza virus A/goose/Guangdong/1/1996 lineage primarily manifest in the lower respiratory tract [10] and are relatively rare, with only 889 cases reported from 23 countries from 2003–April 2024 [11]. To place the recorded H5N1 cases in context, the U.S. CDC estimates that seasonal influenza caused 31 million cases and 21 thousand deaths in the U.S. alone during the 2022–2023 flu season [12]. Despite the low historical reported incidence of H5N1 in humans, the subtype remains a public health concern as 52% of the 889 reported cases outside the U.S. resulted in death [11]. Transmission to humans is primarily through exposure to infected animals, although probable examples of non-sustained, human-to-human transmission have been documented [13–16]. From March 2024 to October 31, 2024, there were 46 human H5N1 cases reported; 25 cases reported exposure to infected cows, 20 cases reported exposure to infected poultry, and 1 case had no identified exposure source [17]. The most common clinical signs reported in the 46 cases are conjunctivitis (93%), fever (49%), and respiratory symptoms (36%) [17].

The detection of H5N1 in milk in the United States (first reported in March 2024 [2]) from cows with and without clinical signs posed an unknown threat to food safety and led to a chain of rapid risk management initiatives and data gathering efforts. As of September 18, 2024, no viable H5N1 virus had been detected in U.S. retail milk samples [18], and the federal-state public health system surveillance had not identified any foodborne H5N1 illnesses in humans [19]. To inform early and ongoing decision making during the outbreak in dairy cows, the FDA quickly leveraged risk assessment methods and tools. Over a five-month timeframe, we developed four iterative versions of the risk assessment model using new data and information to help refine the exposure and risk estimates for milk. Here, we report findings from these efforts and the risk assessment approaches employed to explore the impact of a range of assumptions and scenarios in a novel, data-scarce situation. This work identifies key data gaps and uncertainties for future H5N1 research and presents a framework that can be quickly applied in the event of future H5N1 or other emerging pathogens of potential concerns in foods.

## Materials and methods

The risks of illness associated with raw and pasteurized milk consumption during the H5N1 outbreak in dairy cows were characterized with two concurrent methods: a quantitative risk assessment (QRA) and a surveillance strategy analysis. The QRA calculated risk of illness based on the integration of available data on virus levels in milk, consumption of milk, and dose response, termed a “bottom-up” approach [20,21] hereafter. The bottom-up approach estimated potential cases of illness and facilitated the evaluation of a variety of intervention options. Concurrently, knowledge about the current U.S. influenza surveillance system was used to estimate the number of cases that could be detected, termed a “top-down” approach hereafter. The top-down approach provided a measure of risk based on current epidemiological data to compare with the risk estimate from the bottom-up approach (i.e., results from the two approaches are mutually informative). This comparison provided context for the interpretation of epidemiological data and results from the bottom-up risk scenarios with highly uncertain parameters by characterizing the upper bound number of cases (i.e., to anchor the risk assessment).

### Bottom-up exposure assessment

FDA conducted the QRA using the best available data to estimate the risk of illness associated with potential H5N1 presence in milk and evaluate the public health impact of potential control measures to help identify optimal risk management strategies. The QRA, besides hazard identification, included three main components: exposure assessment, hazard characterization (dose response analysis), and risk characterization. We quickly developed the QRA with the main components and used the model to evaluate a range of iterative risk scenarios to accommodate rapidly evolving data availability from the initial incident alert to the current state of the science. The evolution of data availability and how we refined the exposure assessment and dose response analysis, and subsequent risk characterization, is outlined for each step.

#### H5N1 in raw milk percent positive estimates.

Preliminary H5N1 percent positive estimates were obtained from 275 double-blinded bulk tank raw milk samples received at the testing lab during April 18–27, 2024 [22]. Samples were drawn from H5N1 herd positive states (targeted sampling) in regions known to include impacted farms and thus were not expected to be representative of H5N1 in bulk milk tanks in the U.S. or in the impacted states. The first iteration of the QRA, version 1.0 (April 2024), had inputs for contamination based on reported detection of the virus in a limited number of samples from affected dairy farms that was a subset of the samples eventually published in Spackman et al. [22]. We updated the model to version 1.1 (May 2024) using sampling data (S1 Table, risk scenario A1) of the percent positive of RT-qPCR (reverse transcription quantitative polymerase chain reaction) for samples with a valid test result (n = 265). In version 1.1, we assumed that the national U.S. milk supply, before pasteurization, shared the same H5N1 percent positive as the 265 samples. This assumption likely led to overestimating the occurrence of H5N1 in bulk tank raw milk and associated risk estimates, even though it served as a starting point in the risk assessment in an effort to capture a worst-case scenario during the early stages of the outbreak investigation. The estimated percent positive of H5N1 in bulk tank raw milk samples was 59.6% [22].

While the preliminary estimates of H5N1 percent positive relied on presumptive positive RT-qPCR results, estimates of viable virus presence, via propagation in chicken eggs, became available from the previously tested bulk tank raw milk samples [22]. Provided that viral nucleic material can be amplified even in the absence of viable (infectious) virus, in version 1.2 (June 2024) we updated our H5N1 percent positive estimates based on viable virus positive samples. Consistent with the contamination inputs in version 1.1, the percent positive of viable H5N1 in these samples was assumed for the U.S. milk supply before pasteurization. In version 1.2, the estimated percent positive of H5N1 in bulk tank raw milk samples was 14.8% (S1 Table, risk scenario A2).

We updated the model again to version 1.3 (July 2024), which incorporated production weighting to better estimate the fraction of the U.S. milk supply that might test positive for H5N1. To achieve this, the proportion each U.S. state represented of the total U.S. milk production was taken from USDA estimates [23]. The production weighted percent positive was the product of the H5N1 percent positive in bulk tank raw milk samples by the percent of the total milk production from states which at any point yielded H5N1 positive herds. In version 1.3, the estimated percent positive of H5N1 in bulk tank raw milk samples was 5.8% (S1 Table, risk scenario A3).

#### H5N1 concentration distributions.

Bulk tank raw milk samples that tested positive for H5N1 by RT-qPCR were subsequently quantified by culturing viable virus in embryonating chicken eggs [22]. The RT-qPCR results for the bulk tank samples were available more rapidly than the egg culture results and, accordingly, the QRA version 1.1 assumed a positive rate of 59.6% in the bulk tank samples with an empirical distribution ranging from 1.20 to 8.02 log_10_ RT-qPCR titer/mL. Early egg culture findings showed that levels of infectious virus were lower than the viral RNA concentrations, but the difference varied between 0.7 and 6.0 log_10_ with no clear pattern across the paired RT-qPCR titer and viable virus level across all samples [22]. As such, a uniform distribution of 0.7 to 6 log_10_ reduction was applied to estimate the concentration of viable virus in the bulk tank samples (S1 Table, risk scenario A1). After the egg culture results were complete, it was evident that there was minimal association between the estimated titer by RT-qPCR and quantified viable virus [22]. The QRA was updated to include a concentration distribution ranging from 1.0 to 6.3 log_10_ EID_50_/mL from the egg culture results with a positive rate of 14.8% or 5.8% (S1 Table, risk scenario A2 or A3).

#### Consumption.

The number of daily eating occasions per capita was estimated using data from the National Health and Nutrition Examination Survey (NHANES), specifically the food consumption portion of NHANES, What We Eat in America (WWEIA), from the years 2017–2018 [24]. NHANES/WWEIA collects data on consumption patterns via two-day dietary recall surveys. The analyses included milk consumed as a beverage (including flavored milks and milk added to hot beverages), using data for respondents who provided food intake information for both NHANES/WWEIA survey days (i.e., data respondents who provided only food intake information for only one day were excluded from analyses). NHANES/WWEIA recommended statistical weights for two-day dietary respondents were used in the analyses. The number of daily eating occasions per capita was calculated as the total weighted number of eating occasions divided by the weighted number of survey participants, and then divided by two, as there were two days of the survey. Eating occasions were defined by a 15-minute minimum elapsed time since last consumption of the food. Based on data from NHANES/WWEIA 2017–2018 for the total population, we estimated 0.45 eating occasions per day of milk is consumed as a beverage per capita in the United States. Multiplying the per capita consumption rate by the 2023 U.S. census estimate of the U.S. population (n = 334,914,895 persons) yielded 150,711,703 daily fluid milk eating occasions (i.e., servings) in the United States.

The pasteurization status of milk products consumed is not measured by the NHANES surveys. To estimate the number of raw fluid milk servings eaten in the U.S., we queried the Foodborne Disease Active Surveillance Network (FoodNet) Population Survey 2018–2019 data [25] for the proportion of consumers who ate raw or unpasteurized milk (2.0%) in the last seven days (“In the past 7 days, did you/your child eat unpasteurized or raw milk from any animal?”). The FoodNet survey data has been used previously to estimate exposure to raw milk in risk assessments [26]. To account for the increased survey recall period in the FoodNet study, we constructed an adjustment factor by dividing the proportion of consumers in FoodNet (both raw and pasteurized milk) by the proportion of consumers in NHANES for raw milk. The proportion of consumers in the FoodNet survey was then adjusted by dividing the estimate by the adjustment factor. Using the adjustment factor, we estimated that ~1.2% of U.S. consumers regularly consume raw or unpasteurized milk. Our estimate of raw milk consumers was in line with a previous study, which had estimated that approximately 1% of U.S. adults consume raw milk at least once a week [27]. To estimate the number of raw milk servings, we assumed that the proportion of raw milk consumers represented the percent of total servings in NHANES which are consumed raw (raw milk servings = total servings × percent raw milk consumers), raw milk consumers do not consume pasteurized milk, and that the total fluid milk servings in the U.S. is the summation of pasteurized and raw milk products. With these assumptions, we estimated that 1,808,540 raw milk servings and 148,903,163 pasteurized milk servings are consumed daily in the United States.

Serving size variability was modeled from the NHANES survey data using an empirical distribution (S2 Table). We assumed that serving size variability was identical between pasteurized and raw milk servings. The parameters for pasteurized and raw milk consumption reflected a high level of certainty from the beginning of the QRA, and minimal changes were made as the QRA evolved from the initial incident alert.

#### Pasteurization efficacy.

During the development of the QRA version 1.0, there were no H5N1-specific inactivation studies in dairy products. Initial risk estimates were informed by pasteurization efficacy data for other viruses and final risk estimates were informed by pilot-scale continuous flow pasteurization experiments [22]. Typical pasteurization conditions are termed Low Temperature Long Time (LTLT) for 63°C for 30 min and High Temperature Short Time (HTST) for 72°C for 15 seconds [28].

In lieu of H5N1 specific data for milk, the inactivation of non-enveloped viruses was considered a lower-bound estimate for the enveloped H5N1, as non-enveloped viruses are typically considered more resistant to environmental stressors than enveloped viruses. The identified experiments suggested that a non-enveloped virus would likely be reduced by at least 3-log_10_ reduction [29–32] under milk pasteurization conditions but an enveloped virus may be reduced by more than 5-log_10_ reduction [33–35]. Based on these findings, in the QRA version 1.1, we developed a risk scenario with a uniform distribution of 3–4-log_10_ reduction for pasteurization efficacy (S2 Table) to ensure initial conservative estimates of risk (Table 1).

**Table 1:** Evolution of data availability, reliability, and relevance for the pasteurized milk consumption QRA from version 1.0 (April) to updated versions in May to July 2024.

Parameter	Version 1.1 (May)	Version 1.2 (June)	Version 1.3 (July)	References (examples)
Percent -Positive	59.6% (158/265) RT-qPCR	14.8% (39/264) Egg Propagation	5.8% Egg Propagation Production Weighted	Spackman et al., 2024a
Concentration	Empirical Dist. for RT-qPCR titer (Min: 1.2, 50th: 3.9, Max 8.03 log_10_/mL) reduced by Uniform (0.7, 6) log_10 _reduction	Empirical Dist. for EID_50_ (Min: 1.0, 50th: 3.5, Max: 6.3 log_10_/mL)	Empirical Dist. for EID_50_ (Min: 1.0, 50th: 3.5, Max: 6.3 log_10_/mL)	Spackman et al., 2024a
Pasteurization	Uniform (3, 4)^a^ log_10 _reduction	Approximately^a^ 7 log_10_ reduction	Uniform (12, 13)^b^ log_10_ reduction at least	**a**: Hewitt et al., 2009; Parry & Mortimer, 1984; Bidawid et al., 2000; Pitino et al., 2021; **b**: Spackman et al., 2024a
Partitioning Into Containers	1890 g	1890 g	1890 g	Half-gallon mass
Consumption	149x10^6^ servings/day Serving size: Empirical distribution see S2 Table	149x10^6^ servings/day Serving size: Empirical distribution see S2 Table	149x10^6^ servings/day Serving size: Empirical distribution see S2 Table	CDC, 2020; CDC 2022
Dose Response	Ingestion: r = 1.35x10^−12^; Inhalation: r = 1.35x10^−9^	Ingestion: r = 1.35x10^−12^; Inhalation: r = 1.35x10^−9^	Ingestion: r = 1.35x10^−12^; Inhalation: r = 1.35x10^−9^	U.S. FSIS-FDA-APHIS, 2010; Belser et al., 2011; CDC, 2024b; Eisfeld et al., 2024; Hirose et al., 2017; Lipatov et al., 2008
Predicted Cases per Day	0.39 [0.07, 3.41]	7.60x10^−5^ [1.35x10^−5^, 4.70x10^−4^]	1.19x10^−10^ [2.11x10^−^11, 1.05x10^−9^]	

During April 18 to April 22, 2024, there were 297 retail samples of milk and milk-based products collected and analyzed as part of the FDA’s pilot sampling study in coordination with USDA [36]. Overall, there were 60 positive RT-qPCR detections of viral RNA in the 297 samples (20%). Although viral RNA was detected in 60 samples, no samples had viable virus by egg inoculation studies, further indicating that H5N1 was sufficiently heat sensitive and that our original estimate of 3–4-log_10_ reduction during pasteurization was likely an underestimate.

We used the QRA to evaluate a range of risk scenarios to represent what-if pasteurization efficiency was incremental from 3 to 10-log_10_ reduction. As more H5N1-specific thermal resistance experiments were published, our assessment of the risk scenario in the QRA most representative of pasteurized milk was updated accordingly. A study [37] evaluated the effect of HTST pasteurization using thermocycler tubes containing wild-type H5N1 (isolated from chicken) inoculated in raw milk with an inoculum of 3 × 10^7^ EID_50_ (volume not reported). Viable virus was not detected by egg inoculation assays (limit of detection not reported) after the heat treatment, suggesting that more than 7-log_10_ reduction might occur from HTST conditions, under an assumption of a limit of detection of 0 log_10_ EID_50_. This finding was supported by another set of benchtop experiments in which heat treatment at 72 °C for 15 seconds resulted in at least 7-log_10_ reduction of avian H5N1 in raw milk, where the inoculum was approximately 7 log_10_ EID_50_/mL (highest inoculum 7.75 log_10_) [38]. Investigators detected no viable virus after the heat treatment by egg inoculation assays (limit of detection ~ 0.5 log_10_ EID_50_/mL). There was some uncertainty in the estimated degree of virus reduction because some of these studies [37,38] were performed using samples in PCR tubes heated in a thermocycler, an experimental design that in general could have issues with precise temperature control [39] leading to possible exposure of the virus to temperatures above the target temperature in the thermocycler block. In version 1.2, preliminary results from at least three runs of a thermal inactivation study using pilot-scale continuous flow pasteurization equipment showed at least a 7-log_10_ reduction of H5N1 in raw milk, and the risk assessment was updated accordingly. The thermal inactivation study with pilot-scale continuous flow pasteurization equipment was completed and publicly shared in July [22]. Data from the completed study showed more than 5.8-log_10_ reduction of H5N1 in spiked homogenized raw milk while heating to the process target temperature of 72.5°C before the holding tube [22]. It was estimated that more than 12-log_10_ reduction would occur during the holding time of 15 seconds at 72.5°C [22]. We added a new risk scenario in the most updated risk model, version 1.3 (Table 1), where we applied a uniform variability distribution for pasteurization efficacy, with values ranging from 12–13 log_10_ reduction (S2 Table).

### Hazard characterization

A 2024 study on pathogenicity and transmissibility of bovine H5N1 in mice and ferrets [40] showed that H5N1 binds to both the upper and lower respiratory tracts of those animals and spreads to non-respiratory organs upon infections. Eisfeld et al. also showed that in mice orally inoculated with the same H5N1 in milk at 1.3 to 3.25 x 10^3^ PFU, 40–80% of the mice had systemic infections, compared to a mouse lethal dose 50 (LD_50_) of 31.6 PFU after intranasal inoculation [40]. This suggests consumption dose response might be 100 times less sensitive than intranasal dose response in mice. Furthermore, intranasal inoculation in mice (10^3^ PFU) and ferrets (10^6^ PFU) of the same bovine H5N1 resulted in similar systemic infections, which suggest there might be up to 1000 times inter-species differences in sensitivity given the same intranasal dose. Importantly, ferret models are considered the best small mammal model for evaluating respiratory influenza virus infection and transmission in humans, as ferrets and human share similar lung physiology and have the necessary receptors for influenza viruses distributed throughout the respiratory tract [41,42]. The gastrointestinal tracts of pigs, however, are more similar to humans than ferrets, making a pig model potentially more suitable for evaluating oral exposures (potential foodborne infections) [43,44].

Transmission of influenza viruses is strongly influenced by the capacity of the viral haemagglutinin to bind α2,3-linked and α2,6-linked sialic acid attached to the glycan receptors in epithelial cells in the respiratory tracts [45–47]. Avian influenza viruses exhibit preferential binding to α2,3-linked sialic acid receptors abundant in waterfowls and other animals [48–51] but limited or weak binding to α2,6-linked sialic acid receptors abundant in the human upper respiratory tract. Furthermore, compared with the upper respiratory tract there is a higher proportion of α2,3-linked sialic acid receptors in the human lower respiratory tract [45], to which avian H5N1 viruses may bind and result in severe pneumonia [49,50]. Of note, Eisfeld et al. (2024) showed that bovine H5N1 virus bound to both α2,3- and α2,6-linked sialic acid receptors in vitro, suggesting the capacity to bind to cells in both the upper and the low respiratory tracts in humans. A recent study on the exposure of monkeys to bovine H5N1 virus [52] showed that infections occurred in both the lower and upper respiratory tracts (from intratracheal and intranasal inoculation, respectively) with observed systemic virus spread and pneumonia. In contrast, orogastric exposure resulted in limited virus spread and subclinical infections in the monkeys, which suggests that infection from exposure through contaminated foods is possible but may be insufficient in causing disease compared to direct exposure of the respiratory tract.

Overall, there is a lack of sufficient understanding of host specificity to translate probability of illness data from animal models to probabilities of human illness. Therefore, we chose to adapt the dose response model for inhalation from the interagency risk assessment [43], which was based on human data.

#### Inhalation dose response.

We used an available human dose response model for H5N1 [43] for the intranasal route of exposure, which is an exponential model (Pr(illness|Dose = *D*) = 1-exp(-*r* × *D*)) with an *r* value of 1.35 × 10^−9^ (uncertainty range: 2.42 × 10^−10^ to 1.19 × 10^−8^). In this model, the probability of a response at dose *D* is dependent on the likelihood *r* that a single infectious virus survives to initiate infection in the tissue of concern, and the *r* value of 1.35 × 10^−9^ predicts that it needs an intranasal dose of 8.7 log_10_ EID_50_ to infect 50% of individuals.

An exposure scenario relevant for inhalation and milk consumption is aspiration, where milk positive for H5N1 is misdirected into the airway when an aspiration event occurs during swallowing. Aspiration is relatively rare in healthy adults and tends to be of a trace amount (<1 mL) when it does occur [53,54]. However, certain subpopulations are more at risk for frequent aspiration during consumption of beverages, such as the elderly, infants (especially premature infants), people with dysphagia, and other vulnerable populations [53–55]. In the U.S, approximately 4% of adults report dysphagia (i.e., issues with swallowing) [56]. While there are some methods to assess remaining residue in the subglottic region which may be indicative of relative amount of aspirate that enters airway due to aspiration [57], there are no reports of measured aspirate volume. In the QRA, a 0.1–1 mL equivalent was assumed for the volume of aspirate for model input (S2 Table). Studies assessing swallowing using diagnostic methods such as flexible endoscopic examination of swallowing or video fluoroscopic examination of swallowing have reported aspiration prevalence among participants. These studies are typically conducted on individuals recruited because of suspected dysphagia [58], but have also been conducted on healthy adults [59]. A relatively large study including 3000 participants recruited as inpatients at a hospital reported aspiration prevalence of 22% [60]. Other studies have been conducted with fewer participants, with reported aspiration prevalence ranging from 8% to 68% [58]. We considered two potential scenarios for frequency of aspiration: (1) either a range of 10–30% of all consumption occasions result in some aspiration or (2) 4% of U.S. adults have issues with swallowing and among them the frequency of aspiration is 10–30%, which is 0.4%–1.2% of all consumption occasions. An average of 0.8% (uncertainty range 0.4%–1.2%) was used to determine the aspirated occasions (S2 Table). Although there are potential limitations to equating intranasal and inhalation exposure routes, for the aspiration scenarios evaluated herein, we considered the intranasal dose response appropriate to use without further adjustment.

#### Ingestion dose response.

Although influenza viruses are considered respiratory pathogens, global patterns of wild mammal infections with H5N1 following the ingestion of infected birds indicate that ingestion is a relevant route of exposure to consider [61,62]. This assertion is also supported by animal models. Ferret and mice models have exhibited signs of influenza following intragastric as well as intranasal and intraocular routes of exposure [44,63,64]. The pattern of infection was found to differ between exposure routes and intragastric viral exposure was reported to be less efficient in establishing infection than intranasal exposure [63,64]. Evidence suggests that in mammals, ingested H5N1 can disseminate to nondigestive organs through the lymphatic system of the gastrointestinal tract [63], and a high level of H5N1 virus replication in the respiratory tract was detected in mice after oral inoculation of H5N1-conatining milk [40,65]. In the studies the oral route of exposure was often simulated with intragastric inoculations that artificially removed some of the additional barriers for a pathogen to be able to survive to initiate infection. There is no published dose response model for ingestion and, based on available data, the inhalation dose response model is likely to provide an overestimate of the risk for oral exposure to H5N1.

To modify the inhalation dose response model to approximate an ingestion dose response relationship more closely, we considered the impact of gastric digestion on H5N1 inactivation. Previous works have identified an influenza-specific acid mediated inactivation mechanism, where pH levels around 5 trigger an irreversible conformational change in surface-bound hemagglutinin (HA) [66–68]. Lower pH levels in host cell endosomes trigger viral uncoating and expose hydrophobic residues on HA to facilitate endosome-viral envelope fusion [69]. Previous studies have demonstrated that neither the viral capsid [66] nor genetic material [66,68–70] are meaningfully destroyed or damaged by exposure to pH. Thus, rather than a general breakdown of cellular material, there is a targeted conformational change of a membrane-bound influenza protein upon exposure to low pH which dramatically reduces its infectivity (inactivation).

The pH varies dramatically throughout the gastrointestinal system depending on fed state and fasting state, as well as the types of food consumed [71–73]; for example, for humans in fasting state the gastric pH value on average ranges from approximately 1–2 in the fasted state and from 4 to 5 or higher in fed state depending on meal volume and composition due to buffering and dilution effects. It has been long recognized that fluid milk has excellent buffering capacity in pH ranges typically observed during gastric digestion [74]. To estimate the viral inactivation during gastric passage, we combined observations of the temporal pH dynamics after milk consumption, milk gastric emptying observations [75,76] and influenza inactivation at differing pH levels for HPAI [66,68,70,77] and H5N1-specific strains [78–80]. Based on these data, we assumed that at least a 3-log_10_ reduction of viable virus occurs during gastric passage. This assumption was applied by reducing the *r* parameter of the inhalation exponential dose response model, to 1.35 × 10^−12^ (uncertainty range: 2.42 × 10^−13^ to 1.19 × 10^−11^) for the consumption dose response model (S2 Table). We did not consider viral degradation by digestive processes occurring in the small bowel, or the impact of the reductive conditions found in the large bowel on viral attachment and function.

### Risk characterization

We developed the QRA using FDA-iRISK^®^ [81,82]. The QRA characterized risk for pasteurized and raw milk via ingestion and aspiration exposure routes. We used the QRA to explore the impact of several potential interventions (pasteurization, sampling, and on farm control measures) on the potential risk to consumers if survival of viable virus in milk occurred. Sampling and testing raw milk, in particular for raw milk destined for consumption without pasteurization, was evaluated in the QRA, assuming a limit of detection (LOD) of 4.7 to 5.7 log_10_ EID_50_/mL (based on unpublished data) and subsequent removal of higher-titer raw milk from production (S2 Table). The uncertainties associated with the QRA’s parameters were also probed by running alternative scenarios for uncertainty range of the dose response r-value, different H5N1 contamination in bulk tank raw milk (S1 Table), and different pasteurization efficiency (S2 Table).

### Top-down approach

CDC published [83] a probabilistic framework to estimate the likelihood that novel influenza virus cases would be detected under different community and healthcare (urgent care, emergency department, hospital admission, and intensive care unit) testing strategies in the United States. Using the CDC model and parameters, inferences were derived on the minimum average risk per serving of raw or pasteurized milk that would lead to a high likelihood (95%) to observe at least one case in the United States [83]. This estimate decreases every day with no detected cases. Notably, this estimate is the average risk per serving over a certain period, while the actual risk did vary from the day of first occurrence of H5N1 viruses in milk to the end of the 90, 120, and 150-day period.

## Results

### Bottom-up approach: Risk scenarios in the QRA representing evolving data

Table 1 illustrates the risk scenarios we developed in the QRA from version 1.1 to version 1.3 with shading representing the evolution of our assessment of data availability, reliability, and relevance: darker-shade cell for low, lighter-shade cell for medium, and no-shade cell for high. For the QRA version 1.1, estimates of positive rate, concentration, pasteurization efficacy, and dose response were highly uncertain. In version 1.2 and version 1.3 there were improvements in the positive rate, concentration, and pasteurization efficacy estimates due to the rapid implementation of sampling and pasteurization studies [22,36–38]. As the bulk tank raw milk sampling to date was not completely random, the positive rate and the range of concentrations of viable virus in the raw milk supply remains uncertain.

### Bottom-up approach: Version 1.3 estimates for risk of illness for raw and pasteurized milk

Predictions for the risk of illness from the aspiration or ingestion of raw and pasteurized milk from the QRA version 1.3 are summarized in Table 2. The estimated risk (i.e., predicted number of cases per serving) from ingesting pasteurized milk is estimated to be negligible (8E-19). There are approximately 149 million servings consumed per day in the U.S. and the model predicts 1.2x10^−10^ cases per day accordingly. Furthermore, the predicted cases from exposure for subsequent days and months would be according to linear scaling proportional to the number of servings consumed, e.g., 3.57x10^−9^, 1.07x10^−8^, and 1.79 x10^−8^ cases per 30 days, 90 days, and 150 days, respectively. Given the high pasteurization efficacy (>12 log_10_ reduction), the levels of viable virus detected in raw milk, and the assumed ingestion dose response model herein, we would not expect to see even 1 case of illness in the course of a hundred years of consistent consumption of pasteurized milk (for the current H5N1 virus).

**Table 2:** Predicted risk of illness for raw milk and pasteurized milk scenarios (Version 1.3).

Scenario	Predicted Cases per Serving	Predicted Cases per Day
Pasteurized Milk - Aspiration	1.99x10^−18^ [3.54 x10^−19^, 1.76 x10^−17^]	2.38x10^−12^ [4.22 x10^−13^, 2.09 x10^−11^]
Pasteurized Milk – Ingestion	7.98 x10^−19^ [7.03 x10^−18^, 1.42 x10^−19^]	1.19 x10^−10^ [2.11 x10^−11^, 1.05 x10^−9^]
Raw Milk – Aspiration	5.25 x10^−6^ [9.34 x10^−7^, 4.61 x10^−5^]	0.08 [0.01, 0.67]
Raw Milk – Ingestion	2.06 x10^−6^ [3.66 x10^−7^, 1.81 x10^−5^]	3.72 [0.66, 32.7]

If every consumer aspirates the pasteurized milk 0.8% of the time, the predicted number of cases per day is still negligible (2.38x 10^−12^) despite the inhalation pathway being associated with greater infectivity (r = 1.32x10^−9^).

The risk per serving from raw milk consumption, however, is nearly 12 orders of magnitude greater than that from pasteurized milk consumption. There are approximately 1.81 million servings of raw milk consumed per day in the U.S. and a risk per serving of 2.06x10^−6^ is associated with approximately four cases of illness per day. Furthermore, the predicted cases from exposure for a longer time would be according to linear scaling proportional to the number of raw milk servings consumed, e.g., 111.6, 334.8, and 558 cases per 30 days, 90 days, and 150 days, respectively. After a year of consistent daily consumption of raw milk, the risk model predicts 1,350 cases of illness. If aspiration occurs for 0.8% of raw milk consumption servings, the model predicts less than 1 case of illness per day and 28 cases per year. The 12 order of magnitude difference between the risk per serving from pasteurized and raw milk exposure is directly related to the observed pasteurization efficacy. The risk estimates associated with aspiration are based on assuming H5N1 positive rate is 5.8% (S1 Table, scenario A3), aspiration occurs for 0.8% of all milk consumption servings (i.e., eating occasions) and 0.1–1 g of milk is aspirated per easting occasion, along with other model inputs (S2 Table). If we assumed that aspiration occurs for 20% of all consumption eating occasions, the predicted number of cases per day for pasteurized milk aspirations is 5.9 x10^−11^ and 1.9 for raw milk aspirations (700 cases per year are predicted from raw milk aspirations).

#### Impact of intervention.

The safety of milk is principally ensured by effective preventive control measures throughout the food chain, e.g., on farm management to remove sick cows from milk production, pasteurization, and sanitation controls to prevent cross contamination. In the QRA, we evaluated several interventions including ones that take place prior to processing of milk for the human food supply: 1) on farm control measures (such as removing sick cows from milk production, and mandatory testing and restriction for interstate transport of dairy cattle); 2) sampling and testing of (bulk tank) raw milk and subsequent removal of higher-titer milk from the production of milk; and, 3) pasteurization with various efficiencies, representing initial uncertainty in log_10_ reduction at pasteurization time-temperature combinations.

The potential impact of on farm control measures was analyzed by assuming that the proportion of the H5N1-positive bulk tank raw milk would be impacted by H5N1 positive dairy herds. The QRA results presented herein (Table 2) were generated assuming that the bulk tank samples were representative of the bulk tank raw milk in the 12 herd-positive states (as of July 3, 2024), i.e., positive rate of 5.8%, assuming on farm control measures were effective in limiting the spread of the virus beyond the 12 states. The 12 states produce approximately 40% of the nation’s milk supply. If the virus spread to 12 neighboring states and the proportion of the impacted milk supply increased to 64% (HPAI positive rate 9.5%; S1 Table, scenario A4), the model predicted a linear increase in risk of illness from ingesting pasteurized milk, from 1.19x10^−10^ cases per day to 1.94x10^−10^ cases per day. The predicted risk of illness from ingesting raw milk increased from 3.72 cases per day to 6.08 cases per day. New predicted risks of illness per day for suspected changes in positive rates can be approximated by scaling linearly from the positive rates applied herein.

Sampling and testing of bulk tank raw milk was another intervention assessed in the risk model. If every bulk tank was subject to sampling and testing, with subsequent removal of higher-titer raw milk from production (LOD 4.7 to 5.7 log_10_ EID_50_), followed by pasteurization, the model predicted 9.74x10^−12^ cases per day from pasteurized milk ingestion, a reduction of approximately 1.1 orders of magnitude. If high-titer raw milk was sampled, tested, and removed before packaging, the predicted risk from raw milk consumption was 0.30 cases per day (~110 cases per year), a reduction of approximately 1.1 orders of magnitude.

**Fig 1:**
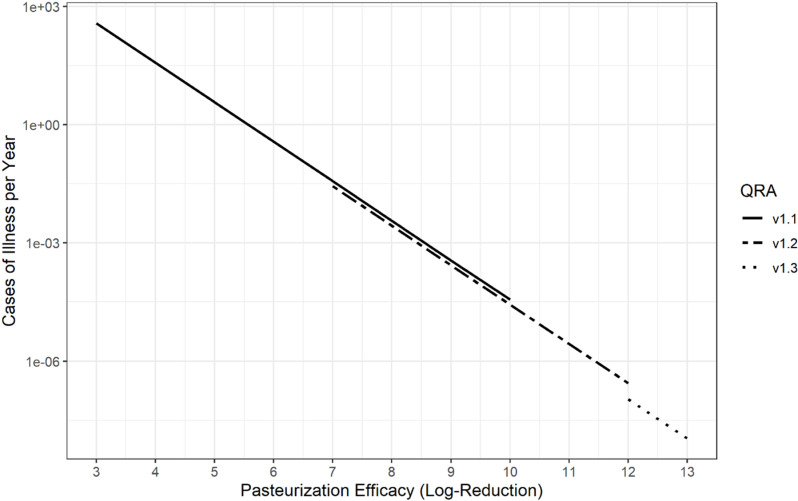
Predicted number of cases per year for increasing pasteurization efficacies for each version of the QRA. In the earlier v1.1 with low amount of data, for pasteurization efficacies of 3 log_10_ and 3 to 4 log_10_ reduction, the predicted number of cases per year was 376.0 (uncertainty range: 69.4 to 3321.5) and 142.4 (uncertainty range: 25.6 to 1244.7), respectively.

It is evident from the QRA that the high pasteurization efficacy (>12 log_10_ reduction) results in extremely low risk for pasteurized milk consumption. Before the most recent pasteurization findings [22], we used the QRA version 1.1 to explore a range of risk scenarios assuming 3–10 log_10_ reduction for pasteurization efficacy. If all other variables are kept the same (concentration in the bulk tank, positive rate, dose response parameter, consumption), the predicted risk of illness scales linearly with pasteurization efficacy as shown in Fig 1. Notably, in the early stages of the outbreak, available thermal inactivation data for HPAI in eggs [84,85] and for non-enveloped viruses in milk [29–33] suggested that at least 5 log_10_ reduction of H5N1 (an envelope virus and more sensitive to heat) would occur during pasteurization. This efficacy was associated with a mean of 0.01 cases per day, and less than 4 cases per year for version 1.1 of the QRA (Fig 1).

For comparison, Fig 1 also shows the predicted cases per year assuming pasteurization efficiency was 7–13 log_10_ reduction and the positive rate and levels of HPAI in bulk tank raw milk were updated to version 1.2 (S1 Table, scenario A2) or version 1.3 (S1 Table, scenario A3).

### Top-down approach: Surveillance strategy expectations and limitations

It is expected that only a minority of all novel influenza cases would be detected by current surveillance efforts. Using the baseline model (novel influenza has similar severity to seasonal influenza) and parameters for urgent care settings specified in Morris et al. [83], the probability of detecting through this system each case of novel influenza was estimated to be 1.29% (CI95%: 0.58%, 2.61%), where the uncertainty was linked to the uncertainty in parameter estimates in Morris et al. [83]. From this estimate, we evaluated the minimal incidence rate that would be detected (at least one confirmed case) with a probability of 95% to be 0.71 (CI95%: 0.35, 1.57) cases per million, corresponding to 233 cases in the U.S. population (CI95%: 115; 516 cases). We did not consider any potential geographical (state-to-state, rural-to-urban) variability in the aptitude of the surveillance system to detect a case because Morris et al. (2024) provides a single estimate for each scenario.

This baseline model is conservative (assuming the novel influenza has similar severity to seasonal influenza) if compared to alternative models developed in Morris et al. (2024) that include increased severity scenarios. If the severity of illness is assumed to be similar to recent H5-like viruses, there would be an increased proportion of symptomatic individuals and a higher proportion of symptomatic individuals seeking care compared to the baseline, following the assumptions and parameterization in Morris et al. (2024). Facing a virus with severity close to recent H5-like viruses, the probability to detect each case would be higher, and would be higher in hospital (8.79%, CI95%: 4.58%, 15.10%) and ICU (6.08%, CI95%: 2.96, 10.82%) settings than in urgent care settings (2.81%, CI95%: 1.29, 5.46%). From the hospital setting estimates, we evaluated the minimal incidence rate that would be detected (at least one confirmed case) with a probability of 95% to be 0.10 (CI95%: 0.06, 0.20) cases per million, corresponding to 34 (CI95%: 20; 65) cases in the U.S. population. Notably, if the virus evolved to exhibit efficient human-to-human transmission, this more efficient transmission and the presence of clusters around cases would similarly make the cases easier to detect. Similarly, if the mortality rate of H5N1 infection happened to be as high as the one observed in other countries (>50%), the cases would be detected with even a much higher probability.

**Fig 2:**
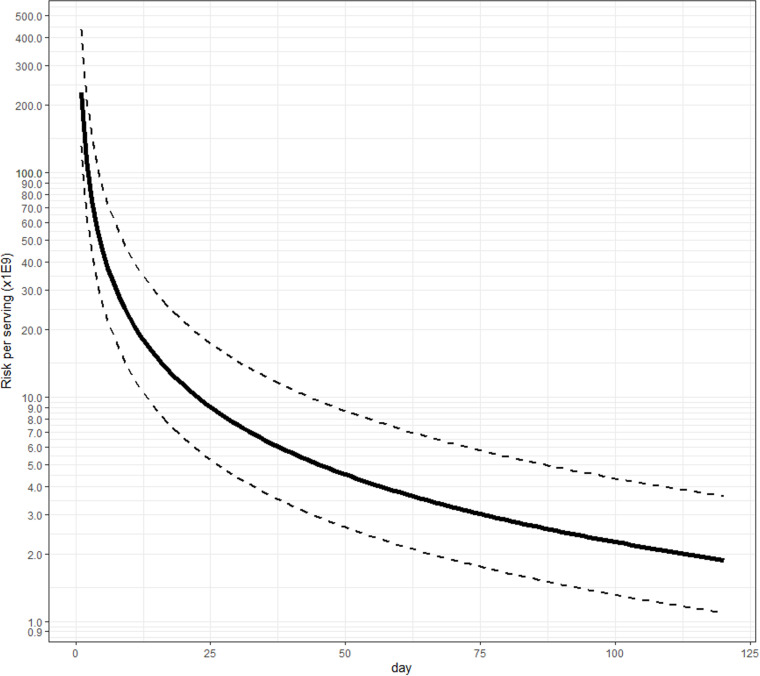
Minimal average risk per serving of pasteurized milk that would lead to a 95% chance of having observed at least one case, as a function of the number of days of the presence of H5N1 in milk (H5-like virus severity, hospital settings estimates).

**Fig 3:**
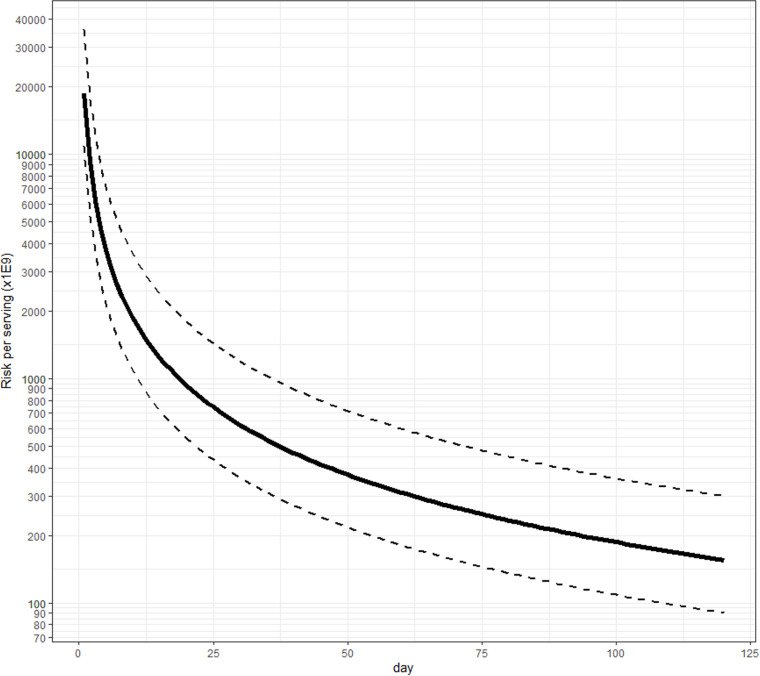
Minimal average risk per serving of raw milk that would lead to a 95% chance of having observed at least one case, as a function of the number of days of the presence of H5N1 in milk (H5-like virus severity, hospital settings estimates).

Using the top-down approach, we made inferences on the minimal average risk per serving of pasteurized milk (Fig 2) or per serving of raw milk (Fig 3) that should lead to at least one *observed* case in the U.S. with a probability >0.95. Assuming 151 million servings of pasteurized milk per day, this estimate decreases every day without any reported case (Table 3). We estimated 0.051 (CI95% in Table 3) cases per million after 30 days, 0.026 after 60 days, and 0.017 after 90 days considering an H5N1 virus with severity similar to seasonal influenza and urgent care settings. Under the assumption of an H5-like virus and using hospital settings, we estimated 0.00753 cases per million after 30 days, 0.00376 after 60 days, and 0.00251 after 90 days. Given the risk of illness for pasteurized milk exposure estimated by the bottom-up approach (2.00 × 10^−18^ to 8.00 × 10^−19^), we would not expect to detect a case in the U.S. if the severity of illness is in line with the assumption from Morris et al. (2024). The estimates are inversely proportional to the number of days without observed cases: e.g., the minimal average risk per serving of milk that should lead to at least one observed case in the U.S. after 90 days was predicted to be 1/3 the estimate after 30 days (Table 3, e.g., 0.00753*(1/3) = 0.00251); by extension, after 120 and 150 days, the predicted risk would be 1/4 and 1/5 the estimate after 30 days.

**Table 3:** Top-down approach: Predicted risk for raw milk and pasteurized milk scenarios as minimal average risk (95% CI) per million servings of milk that should lead to at least one *observed* case with a probability >0.95.

Scenario	Minimum risk: 30 days	Minimum risk: 60 days	Minimum risk: 90 days
Pasteurized Milk – seasonal flu severity – urgent care settings	0.051 (0.025, 0.115)	0.026 (0.013, 0.057)	0.017 (0.008, 0.038)
Pasteurized Milk – H5-like severity – hospital settings	0.00753 (0.00439, 0.0145)	0.00376 (0.00219, 0.0723)	0.00251 (0.00146, 0.00482)
Raw Milk – seasonal flu severity – urgent care settings	4.25 (2.09, 9.46)	2.13 (1.05, 4.73)	1.42 (0.70, 3.15)
Raw Milk – H5-like severity – hospital settings	0.622 (0.362, 1.19)	0.311 (0.181, 0.597)	0.207 (0.121, 0.398)

Considering raw milk consumption of an estimated 660 million servings per year (*cf. supra*), the estimate of the minimal average risk per serving of raw milk that would lead to at least one *observed* case in the raw milk consumer population, and using baseline assumptions, we estimated 4.25 cases per million after 30 days, 2.13 after 60 days, and 1.42 (0.70, 3.15) after 90 days as shown in Table 3, with CI95% estimates. If we assumed H5-like virus severity and hospital settings, the estimates were 0.622 cases per million after 30 days, 0.311 after 60 days, and 0.207 after 90 days. Our ability to detect H5N1 illness in consumers of raw milk was estimated to be similar (baseline model, less severe illness) or better (H5-like viruses, more severe illness) than our ability to detect illness among pasteurized milk consumers, assuming the same contamination profile in raw milk. Assuming percent positive and levels in the raw milk supply are similar to bulk tank raw milk, the risk of illness from consumption and aspiration (assuming it occurs in 0.8% of all consumption servings) estimated by the bottom-up approach is 2.1 cases per million, which corresponds to one case being detected in the U.S. after two months of exposure assuming normal flu-like severity estimated by the top-down approach. As of the end of July (five months assuming exposure started in March), there were no cases of H5N1 reported from the ingestion of milk products.

## Discussion

The FDA began assessing the potential risk to U.S. consumers of milk and milk products immediately after the initial alerts of an H5N1 outbreak in dairy cattle. We employed two complementary and parallel approaches, in which a bottom-up prediction of risk per serving and a top-down calculation of the risk based on the observed number of detected cases were carried out simultaneously. The bottom-up QRA was compared to the top-down analysis to provide initial risk estimates and credible bounds and to facilitate the identification and prioritization of future research needs in a data-scarce scenario for an emerging hazard of potential concern to consumers of dairy products.

Risk estimates from our assessment provide insights on the potential magnitude of risk from an emerging pathogen of potential concern in milk through a structured framework to integrate available data on virus prevalence and levels in milk, consumption of milk, and dose response. Importantly, using the QRA framework allowed us to delineate data availability regarding what was known, what was unknown or uncertain, and what explicit assumptions were made in the quantitative assessment. This in turn allowed us to put the potential emerging risk of H5N1 in an order-of-magnitude-wise context when comparing our assessment with other (often qualitative or semi-quantitative) assessments of H5N1 in dairy products conducted by other organizations, and when comparing with predicted risk for known foodborne pathogens in foods. Using the QRA also enabled us to characterize the influences of different factors and to identify which among the many data gaps would be most important to fill. As evident in the evolution of the QRA (Table 1), pasteurization efficacy is the exposure parameter with the greatest impact on risk associated with pasteurized milk. Pasteurization is an established and required control for milk sold in interstate commerce in the U.S. [28] and our preliminary QRA indicated that an efficacy of 5–6-log_10_ reduction would be sufficient to reduce the potential risk associated with pasteurized milk to less than 1 case per year. Any additional log_10_ reduction in pasteurization efficacy would further reduce risk per serving by a factor of 10. Although the literature for other enveloped viruses [33–35] and the absence of viable H5N1 in the 297 RT-qPCR positive retail milk samples [36] indicated that more than 5 log_10_ reduction from pasteurization was likely, validating that assumption was a clear priority. After experiments in a pilot-scale continuous flow pasteurizer led to the determination that more than 12 log_10_ reduction of H5N1 were expected to occur with classic HTST methods [22], the QRA was updated and used to identify other priorities for research.

The confidence in percent positive and concentration of viable virus evolved from low to medium in the QRA (Table 1) because, although sampling studies have been conducted, the studies were not designed to be nationally representative [22]. The sampling data was first available as RT-qPCR titers and later as viable virus (EID_50_) levels. Lactating cows rapidly develop antibodies after infections [36], with some having lower or higher antibody levels present to inactivate the virus depending on the number of days post infection [86]. That a massive range (five orders of magnitude of differences) between the paired RT-qPCR titer and viable virus level was observed across all the samples [22] might suggest the bulk tank raw milk was pooled from cows at a wide range of stages regarding days post infection. In the QRA model v1.1, it was clear that the viable virus level would be expected to be lower than the RT-qPCR titer; thus, an adjustment was warranted. Based on the available data, we applied reductions by Uniform (0.7, 6.0) log_10_ to the RT-qPCR titer values to estimate an equivalent viable virus concentration distribution. In the QRA model v1.2, we further refined the viable virus concentration distribution when the data specific for viable virus became available for the bulk tank raw milk samples. The impact of data limitations was further assessed by simulating changes in the production-weighted percent positive, and it was determined that percent positive had minimal impact on risk while the pasteurization efficacy had more significant impact (i.e., order-of-magnitude impact). These findings suggest that targeted sampling of bulk tank raw milk from affected states to capture the upper bounds of viable virus concentrations would be a higher priority in initial sampling efforts. The data from the sampling informing the distributions of H5N1 concentrations in bulk tank raw milk are based on a limited dataset that is not considered to be nationally representative and are expected to be biased towards milk from infected herds and states with infected herds (and excluded raw milk dairies). As such, the risk estimates presented herein might overestimate risk to consumers. Larger sampling efforts are needed to inform national prevalence and concentration estimates pre-pasteurization (in bulk tank milk), to capture raw milk from asymptomatic and convalescent cows that is typically pooled and comingled from numerous farms before pasteurizing and processing. Analysis should focus on quantifying viable virus. Ideally, representative sampling is needed to elucidate the variability of virus loads in bulk tank samples to appropriately characterize the distribution of viable virus levels, in particular the maximum level and the likelihood of upper levels. Information on viral shedding in cow’s milk throughout the course of H5N1 infections would also be helpful in characterizing the maximum viral concentrations expected and evaluating the time interval needed before convalescing cows can be returned to the milking herd. Data adequate to quantify viable virus concentration is particularly important given that sensitivity analysis in this QRA indicates that the upper tail of the concentration distribution is a key driver for the risk estimate and the estimated relative risk reduction from interventions.

There were no data available for the prevalence and levels of H5N1 in raw milk collected on farms supplying the raw milk retail market or in retail raw milk specifically, and thus to estimate risk it was assumed that the raw milk supply had the same percent positive and concentration of viable H5N1 as had been detected in bulk tank raw milk pre-pasteurization [22]. Under these assumptions, the risk to raw milk consumers was predicted to be at least 12 orders of magnitude greater than the risk to pasteurized milk consumers. According to the top-down approach, this level of risk should have been associated with detected cases from raw milk consumption after two months of exposure (by May 2024). The difference between the lack of observed cases and the predicted number of cases from raw milk could suggest that i) raw milk dairy herds had been relatively protected from the outbreak thus far (lower prevalence, lower concentration) by on farm control measures (such as mandatory testing and restriction for interstate transport of dairy cattle, limited number of cows and limited potential spreading of the virus on a farm producing milk destined for raw milk consumption) or operation-specific characteristics, ii) the probability of a virus surviving to initiate infection in an ingestion exposure may be lower than estimated (r = 1.35x10^−12^) in the dose response model, iii) aspiration occurs less frequently than the 0.8% estimated, and/or iv) there are situation-specific factors not fully characterized in the top-down approach based on Morris et al. [83]. To further reduce future risk, the QRA highlights the potential impact of sampling for raw milk suppliers as a control measure. If raw milk is sampled with an LOD of 4.7 to 5.7 log_10_ EID_50_ and the higher-titer raw milk was subsequently removed from the food supply, it would reduce risk by 92% (more than a factor of 10 reduction) compared to the estimates without sampling. Reductions would be larger for tests with lower LODs. In the absence of pasteurization, frequent testing of cattle and sampling of raw milk may be potential risk mitigation measures to protect raw milk consumers.

The lack of observed human cases from raw milk consumption in the first five months of the dairy cattle outbreak could indicate that the current dose response for H5N1 ingestion (r = 1.35x10^−12^) is an overestimate. The dose response relationship for an ingestion exposure is highly uncertain as illustrated in Table 1, and more dose response data are needed to inform our understanding of the pathogenicity of H5N1 in humans after ingestion without aspiration. The modifications to the pre-existing inhalation dose response [43] to derive the ingestion dose response herein were informed by animal studies demonstrating that intragastric exposures were less efficient at establishing infection than intranasal [44,63] and by a known influenza-specific acid inactivation mechanism [69]. A more nuanced assessment of pre-existing and novel ingestion dose response data for H5N1 is a clear research need, which will also support the evaluation of the impact of future virus mutations or reassortment on infectivity.

In the bottom-up QRA we accounted for both variability and uncertainty. While probabilistic distributions were used to model variability (such as using a distribution to model viable virus level in bulk tank milk or the consumption serving size), alternative scenarios were used to evaluate the impact of uncertainty in parameter values and how key data gaps (by using alternative uncertainty ranges as model inputs) could impact risk estimates.

The methodology we employed allowed for generating risk estimates quickly under a wide range of what-if scenarios, and for the rapid prioritization of research needs in data scarce conditions. The framework and QRA were easily leveraged to incorporate more data and a more probabilistic approach as more data became available and the data quality improved, which in turn, allowed us to generate risk scenarios and risk estimates most representative of the real-world risk associated with pasteurized milk and raw milk. In addition to the aforementioned primary data needs (including larger pre-pasteurization sampling efforts, raw milk herd surveillance and sampling, and ingestion dose response studies), improved estimates of aspiration volume and frequency are a noted data gap. Aspiration is more common in immunocompromised populations, the elderly, and children [53–55], and inhalation is a more efficient pathway for the establishment of H5N1 infection. Improving data on aspiration specific for these subpopulations will improve the accuracy of the risk estimates for these subpopulations, which will be important for protecting public health as the outbreak of H5N1 in dairy cattle continues. This information would also be useful in understanding the potential public health impact for other pathogens for which aspiration may lead to illness.

In conclusion, our work presents a framework for conducting rapid risk assessments to inform risk management decisions for public health. The risk assessment was a part of the Agency’s efforts to address the emerging concerns of HPAI in milk and other dairy products, including in the preliminary stages of data gathering and research. The dual bottom-up and top-down approaches allow for risk estimate benchmarking and research need prioritization. The framework and its dynamic use described herein can be leveraged in the conduct of ongoing and future risk assessments for H5N1 to evaluate other dairy products such as raw milk cheese or other emerging pathogen outbreaks.

## Supporting information

S1 Table Iterations of model inputs and alternative scenarios for H5N1 contamination in bulk tank raw milk. (DOCX)

S2 Table Model inputs for risk assessment of HPAI in raw and pasteurized milk (bottom-up approach). (DOCX)

S3 Table Distribution of virus levels (RT-qPCR titer). (DOCX)

S4 Table Distribution of viable virus levels (EID50). (DOCX)

S5 Table Distribution of serving size (same for pasteurized milk and raw milk). (DOCX)

S1 File Model code for top-down approach.
