# Supplementary material for: Rapid risk assessment to address emerging concerns of HPAI in raw and pasteurized milk
Source: PLoS One. 2025 Jun 4;20(6):e0322948. doi: 10.1371/journal.pone.0322948 (PMC12136469; doi:10.1371/journal.pone.0322948)
Supplement: S1 Table — (DOCX) [file pone.0322948.s001.docx]

**S1 Table. Iterations of model inputs and alternative scenarios for H5N1 contamination in bulk tank raw milk**

| **Scenario** | **Model input** |
| --- | --- |
| A1 | Based on RT-qPCR:  Positive rate = 59.6%, assuming data from bulk tank raw milk samples (Spackman et al., 2024a) applicable to all States.  Distribution of viable virus levels: Empirical distribution (51.3^th^ 4.0 log_10_, 90.5^th^ 6.6 log_10_, 97.5^th^ 7.2 log_10_, max 8.03 log_10_/ml; see full distribution in S3 Table), with viable virus equivalent: reductions by Uniform(0.7, 6) log_10_. |
| A2 | Based on viable virus (EID_50_):  Positive rate =14.8%, assuming data from bulk tank raw milk samples (Spackman et al., 2024a) were representative of the bulk tank milk in the nation’s milk supply.  Distribution of viable virus levels: Empirical distribution (53.8^th^ 3.5 log_10_, 89.7^th^ 5.4 log_10_, 94.9^th^ 5.5 log_10_, max 6.3 log_10_ EID_50_/ml; see full distribution in S4 Table), based on 39 positive samples with enumeration EID_50_ data. |
| A3 | Based on viable virus (EID_50_):  Positive rate = 5.8%, assuming bulk tank raw milk samples were representative of the bulk tank milk in 12 affected States (as of July 3, 2024) that produce 39.4% of the nation’s milk supply (14.77% x 39.4% = 5.8%).  Distribution of viable virus levels: Same as Scenario A2 |
| A4 | Based on viable virus (EID_50_):  Positive rate =9.5%, assuming the virus spread to 12 neighboring states and the proportion of the impacted milk supply were increased to 64.3% of the nation’s milk supply (14.77% x 64.3% = 9.5%).  Distribution of viable virus levels: Same as Scenario A2 |
