# Supplementary material for: Rapid risk assessment to address emerging concerns of HPAI in raw and pasteurized milk
Source: PLoS One. 2025 Jun 4;20(6):e0322948. doi: 10.1371/journal.pone.0322948 (PMC12136469; doi:10.1371/journal.pone.0322948)
Supplement: S2 Table — (DOCX) [file pone.0322948.s002.docx]

**S2 Table. Model inputs for risk assessment of HPAI in raw and pasteurized milk (bottom-up approach)**

| **Model Element** | **Model input** |
| --- | --- |
| Exposure: Contamination Model | Prevalence and levels of the virus, see S1 Table |
|  | Sampling bulk tank milk or retail milk LOD 4.7 to 5.7 log_10_ RT-qPCR titer |
|  | Pasteurization: Uniform (3, 4) log_10_ or Uniform (12, 13) log_10_ reduction |
| Exposure: Consumption Model (or Aspiration) | Number of servings:  Pasteurized milk: 148,903,163 servings per day  Raw milk: 1,808,540 servings per day |
|  | Serving size (same for pasteurized milk and raw milk):  Empirical distribution (average 212g; 25^th^ 104g, 50^th^ 198g, 95^th^ 516g, 97.5^th^ 536g, 99^th^ 734g). See full distribution in S5 Table. |
|  | Aspiration:  Assuming it occurs in 0.4% to 1.2 % (average 0.8%) of all milk consumption servings (eating occasions), and Uniform (0.1, 1) g of milk is aspirated per eating occasion |
| Dose Response Model | Consumption:  Exponential model, baseline r=1.35x10^-12^; uncertainty upper bound r=1.19x10^-11^, lower bound r=2.4x10^-13^ |
|  | Aspiration:  Exponential model, baseline r=1.35x10^-9^; uncertainty upper bound r=1.19x10^-8^, lower bound r=2.4x10^-10^ |
| Risk Characterization | Estimated risk: 1) Predicted cases per serving or per day for pasteurized milk and for raw milk; 2) Predicted cases per eating occasion aspirated, or per day assuming aspiration occurs in 0.8% of all servings per day. |
|  | Impact of interventions on estimated risk:  Alternative scenarios 1) with on farm control measures to minimize spread of HPAI in raw milk supply (Table S1 scenario A4); 2) with sampling of raw milk before consumption; and 3) with pasteurization. |
|  | Uncertainties in estimated risk:  Alternative scenarios 1) with uncertainty range of the dose response r-value; 2) with different initial contamination in bulk tank raw milk (see Table S1); 3) with aspiration occurs for 20% of all consumption eating occasions; and 4) with different pasteurization efficiency of incremental 3 to 10 log_10_ reduction. |
