# Supplementary material for: Rapid risk assessment to address emerging concerns of HPAI in raw and pasteurized milk
Source: PLoS One. 2025 Jun 4;20(6):e0322948. doi: 10.1371/journal.pone.0322948 (PMC12136469; doi:10.1371/journal.pone.0322948)
Supplement: S3 Table — (DOCX) [file pone.0322948.s003.docx]

S3 Table: Distribution of virus levels (RT-qPCR titer)

| Cumulative probability | log_10_ RT-qPCR titer/mL |
| --- | --- |
| 0 | 1.2 |
| 0.012658 | 1.4 |
| 0.01267 | 1.6 |
| 0.018987 | 1.8 |
| 0.025316 | 2 |
| 0.088608 | 2.2 |
| 0.158228 | 2.4 |
| 0.183544 | 2.6 |
| 0.297468 | 2.8 |
| 0.329114 | 3 |
| 0.367089 | 3.2 |
| 0.398734 | 3.4 |
| 0.424051 | 3.6 |
| 0.468354 | 3.8 |
| 0.512658 | 4 |
| 0.525316 | 4.2 |
| 0.531646 | 4.4 |
| 0.544304 | 4.6 |
| 0.556962 | 4.8 |
| 0.588608 | 5 |
| 0.626582 | 5.2 |
| 0.632911 | 5.4 |
| 0.658228 | 5.6 |
| 0.677215 | 5.8 |
| 0.702532 | 6 |
| 0.772152 | 6.2 |
| 0.860759 | 6.4 |
| 0.905063 | 6.6 |
| 0.943038 | 6.8 |
| 0.94305 | 7 |
| 0.974684 | 7.2 |
| 0.987342 | 7.4 |
| 0.98735 | 7.6 |
| 0.98736 | 7.8 |
| 0.993671 | 8 |
| 1 | 8.03 |
