# Supplementary material for: Rapid risk assessment to address emerging concerns of HPAI in raw and pasteurized milk
Source: PLoS One. 2025 Jun 4;20(6):e0322948. doi: 10.1371/journal.pone.0322948 (PMC12136469; doi:10.1371/journal.pone.0322948)
Supplement: S4 Table — (DOCX) [file pone.0322948.s004.docx]

S4 Table. Distribution of viable virus levels (EID_50_)

| Cumulative probability | log_10_ EID_50_/ml |
| --- | --- |
| 0 | 1 |
| 0.021282 | 1.1 |
| 0.041282 | 1.2 |
| 0.051282 | 1.3 |
| 0.0513 | 1.4 |
| 0.179487 | 1.5 |
| 0.17949 | 1.6 |
| 0.205128 | 1.7 |
| 0.20513 | 1.8 |
| 0.20514 | 1.9 |
| 0.20515 | 2 |
| 0.20516 | 2.1 |
| 0.20517 | 2.2 |
| 0.230769 | 2.3 |
| 0.23077 | 2.4 |
| 0.282051 | 2.5 |
| 0.282052 | 2.6 |
| 0.307692 | 2.7 |
| 0.307693 | 2.8 |
| 0.307694 | 2.9 |
| 0.307695 | 3 |
| 0.307696 | 3.1 |
| 0.307697 | 3.2 |
| 0.384615 | 3.3 |
| 0.38462 | 3.4 |
| 0.538462 | 3.5 |
| 0.538462 | 3.6 |
| 0.589744 | 3.7 |
| 0.589744 | 3.8 |
| 0.589745 | 3.9 |
| 0.589746 | 4 |
| 0.589747 | 4.1 |
| 0.589748 | 4.2 |
| 0.74359 | 4.3 |
| 0.74359 | 4.4 |
| 0.846154 | 4.5 |
| 0.846154 | 4.6 |
| 0.846154 | 4.7 |
| 0.846154 | 4.8 |
| 0.846154 | 4.9 |
| 0.846154 | 5 |
| 0.846154 | 5.1 |
| 0.846154 | 5.2 |
| 0.897436 | 5.3 |
| 0.897436 | 5.4 |
| 0.948718 | 5.5 |
| 0.948718 | 5.6 |
| 0.948718 | 5.7 |
| 0.948718 | 5.8 |
| 0.948718 | 5.9 |
| 0.948718 | 6 |
| 0.948718 | 6.1 |
| 0.948718 | 6.2 |
| 1 | 6.3 |
