# Supplementary material for: Rapid risk assessment to address emerging concerns of HPAI in raw and pasteurized milk
Source: PLoS One. 2025 Jun 4;20(6):e0322948. doi: 10.1371/journal.pone.0322948 (PMC12136469; doi:10.1371/journal.pone.0322948)
Supplement: S5 Table — (DOCX) [file pone.0322948.s005.docx]

S5 Table. Distribution of serving size (same for pasteurized milk and raw milk)

| Cumulative probability | Serving size (g) |
| --- | --- |
| 0 | 0 |
| 0.100 | 28.7 |
| 0.200 | 62.0 |
| 0.250 | 104.2 |
| 0.300 | 121.4 |
| 0.400 | 153.4 |
| 0.500 | 198.1 |
| 0.600 | 243.2 |
| 0.700 | 246.4 |
| 0.750 | 275.4 |
| 0.800 | 320.6 |
| 0.900 | 407.9 |
| 0.950 | 515.9 |
| 0.975 | 536.0 |
| 0.990 | 733.6 |
| 0.995 | 888.9 |
| 0.999 | 1403.1 |
| 1.000 | 1952 |
